# Statistical Inference of the Generalized Inverted Exponential Distribution under Joint Progressively Type-II Censoring

**DOI:** 10.3390/e24050576

**Published:** 2022-04-20

**Authors:** Qiyue Chen, Wenhao Gui

**Affiliations:** School of Mathematics and Statistics, Beijing Jiaotong University, Beijing 100044, China; 19271149@bjtu.edu.cn

**Keywords:** generalized inverted exponential distribution, joint progressively type-II censoring scheme, EM algorithm, maximum likelihood estimation, bootstrap method, Bayesian inference, importance sampling, Monte Carlo simulation

## Abstract

In this paper, we study the statistical inference of the generalized inverted exponential distribution with the same scale parameter and various shape parameters based on joint progressively type-II censored data. The expectation maximization (EM) algorithm is applied to calculate the maximum likelihood estimates (MLEs) of the parameters. We obtain the observed information matrix based on the missing value principle. Interval estimations are computed by the bootstrap method. We provide Bayesian inference for the informative prior and the non-informative prior. The importance sampling technique is performed to derive the Bayesian estimates and credible intervals under the squared error loss function and the linex loss function, respectively. Eventually, we conduct the Monte Carlo simulation and real data analysis. Moreover, we consider the parameters that have order restrictions and provide the maximum likelihood estimates and Bayesian inference.

## 1. Introduction

### 1.1. Generalized Inverted Exponential Distribution

The generalized inverted exponential distribution (GIED) is a modification of the inverse exponential distribution (IED). In this way, it can fit the lifetime data better. The GIED was introduced by [1]. The distribution has a non-constant hazard rate function, which is unimodal and positively skewed. Due to these properties, the distribution can model different shapes of failure rates of aging criteria. Reference [2] proposed the method of the maximum product of spacings for the point estimation of the parameter of the GIED. In [3], acceptance sampling plans were developed based on truncated lifetimes when the life of an item follows a generalized inverted exponential distribution. Reference [4] performed a Monte Carlo simulation for the GIED to analyze the performance of the estimations. Reference [5] studied the point estimation of the parameters of the GIED when the test units are progressively type-II censored. Reference [6] generated samples from the GIED and computed the Bayes estimates. Reference [7] investigated the MLEs of the GIED when the test units are progressively type-II censored. Reference [8] proposed a two-stage group acceptance sampling plan for the GIED under a truncated life experiment.

Provided that *X* is a variable and it is subject to the GIED, the following is the form of the corresponding probability density function (pdf), the cumulative distribution function, as well as hazard function. Here, λ is the shape parameter and θ is the scale parameter. Besides, they are both positive.

(1) $$\begin{array}{c} f\left(x;\theta ,\lambda \right)=\theta \lambda e^{-\frac{\lambda }{x}}x^{-2}{\left(1-e^{-\frac{\lambda }{x}}\right)}^{\theta -1},\;x>0 \end{array}$$

(2) $$\begin{array}{c} F\left(x;\theta ,\lambda \right)=1-{\left(1-e^{-\frac{\lambda }{x}}\right)}^{\theta },\;x>0 \end{array}$$

(3) $$\begin{array}{c} h\left(x;\theta ,\lambda \right)=\theta \lambda e^{-\frac{\lambda }{x}}x^{-2}{\left(1-e^{-\frac{\lambda }{x}}\right)}^{-1},\;x>0 \end{array}$$

**Figure 1:**
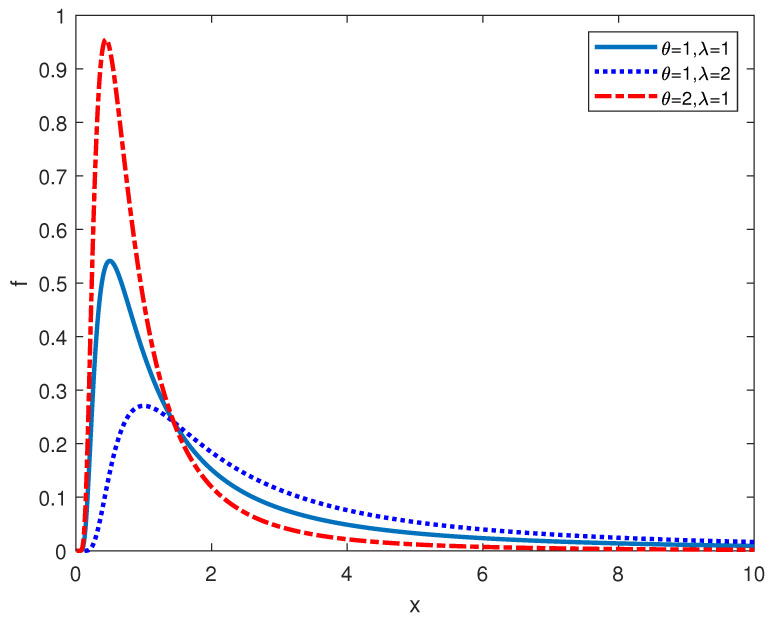
pdf of GIED.

**Figure 2:**
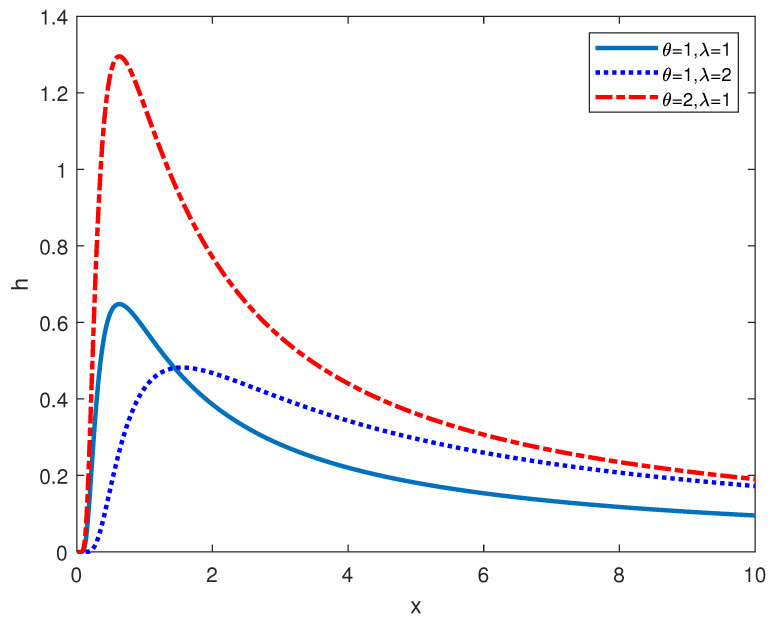
Hazard function of GIED.

The plots of the pdf and hazard function are presented in Figure 1 and Figure 2.

### 1.2. The Joint Progressive Type-II Censoring Scheme

It is difficult to obtain the lifetime data of all the products given the cost and time in many practical situations. Hence, testing experiment censoring is of great importance. A great deal of work has been performed on a variety of censoring schemes. The experimental units cannot be withdrawn during the experiment under the type-I censoring scheme and type-II censoring scheme. Reference [9] described the progressive type-II censoring scheme, in which some units are allowed to be withdrawn during the test. Afterward, we describe the progressive type-II censoring briefly. Suppose *n* units are placed in a lifetime test. *k* is the effective sample size. It also represents the number of observed failures that satisfies k<n. $R_{1}$, ⋯, $R_{k}$ stand for the number of units to be withdrawn for each failure time. Furthermore, they are non-negative integers and satisfy $\sum_{i=1}^{k}\left(R_{i}+1\right)=n$. At the first failure time, $R_{1}$ units are withdrawn from the remaining n−1 surviving units randomly. When the second failure happens, we randomly withdraw $R_{2}$ units from the remaining $n-2-R_{1}$ surviving units. Analogously, when the *k*-th failure happens, the remaining $R_{k}$ surviving units are withdrawn randomly and the test ceases. Reference [10] provided an amount of work about the progressive censoring schemes. Reference [11] dealt with the Bayesian inference on step stress partially accelerated life tests using type-II progressive censored data in the presence of competing failure causes.

**Figure 3:**
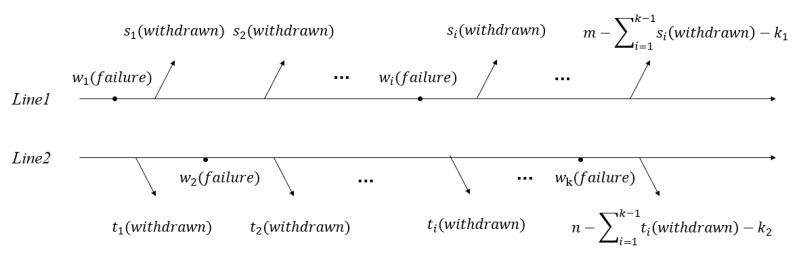
JPC scheme.

Much research about progressive censoring schemes for one sample has been performed by many scholars. However, there is little research on two samples. Reference [12] first introduced the joint progressive censoring schemes for two samples. It is particularly beneficial to compare the life distribution of different products produced by two different assembly production lines on diverse equipment under the same environmental conditions. The joint progressively type-II censoring (JPC) scheme can be briefly described as follows. Suppose the samples are from two different lines, Line 1 and Line 2. The size of the samples of products in Line 1 is *m* and in Line 2 is *n*. Two samples are combined in the joint progressive censoring scheme, and they are placed on a lifetime test. Suppose N=m+n is the size of combined samples. $R_{1}$, ⋯, $R_{k}$ stand for the number of units to be withdrawn in each failure time. Additionally, they are non-negative integers and satisfy $\sum_{i=1}^{k}\left(R_{i}+1\right)=n$. At the first point of failure $w_{1}$, $R_{1}$ units are removed from the combined samples at random. $R_{1}$ units consist of $s_{1}$ units from Line 1 and $t_{1}$ units from Line 2. On the second failure $w_{2}$, $R_{2}$ units are withdrawn from the remaining $m+n-2-R_{1}$ samples at random. $R_{2}$ units consist of $s_{2}$ units from Line 1 and $t_{2}$ units from Line 2. Analogously, at the *k*-th failure time point $w_{k}$, the remaining $R_{k}$ surviving units are withdrawn randomly and the test ceases. Let $z_{1}$, ⋯, $z_{k}$ be random variables. If the *i*-th failure is from Line 1, let $z_{i}=1$. Otherwise, let $z_{i}=0$. Suppose that the censored sample is $\left(\left(w_{1},z_{1},s_{1}\right),\cdots ,\left(w_{k},z_{k},s_{k}\right)\right)$. Here, we introduce $k_{1}=\sum_{i=1}^{k}z_{i}$, which means the number of failures from Line 1. Similarly, $k_{2}=\sum_{i=1}^{k}\left(1-z_{i}\right)=k-k_{1}$, which stands for the number of failures from Line 2. Figure 3 shows the scheme.

Reference [12] applied Bayesian estimation techniques to two exponential distributions for the JPC scheme. Reference [13] considered the JPC scheme for more than two exponential populations and studied the statistical inference. Reference [14] investigated the conditional maximum likelihood estimations and the interval estimations of the Weibull distribution for the JPC scheme. Reference [15] discussed the point estimation and obtained the confidence intervals of two Weibull populations for the JPC scheme. Reference [16] obtained the Bayes estimation when data were sampled in the JPC scheme from a general class of distributions. Reference [17] studied the expected number of failures in the lifetime tests under the JPC scheme for various distributions. Besides, a new type-II progressive censoring scheme for two groups was introduced by [18]. Reference [19] extended the JPC scheme for multiple exponential populations and studied the statistical inference. Reference [20] studied the likelihood and Bayesian inference when data were sampled in the JPC scheme from the GIED.

A few scholars have studied the statistical inference of the generalized inverted exponential distribution when the test units are progressively type-II censored. However, no one has studied the statistical inference of the generalized inverted exponential distribution under joint progressively type-II censoring. The research on this aspect is of great significance.

In this article, we provide statistical inference and study the JPC scheme for two groups that follow the GIED with the same scale parameter. The expectation maximization algorithm is adopted to calculate the maximum likelihood estimates of the parameters. In light of the missing value principle, we derive the observed information matrix. We obtain the interval estimations by the bootstrap-p method based on the Fisher information matrix. We assume a Gamma prior for the shape and scale parameters. The Bayes estimates and credible intervals for the informative prior and the non-informative prior under the linex loss function and squared error loss function are calculated by adopting the importance sampling technique. The performances of various methods are compared through the Monte Carlo simulation. Besides, we conduct real data analysis. Moreover, in many practical cases, the experimenters may know that the lifetime of various populations is orderly. We investigate the problem that the parameters have order restrictions. We discuss the maximum likelihood estimation and Bayesian inference of the parameters.

The rest of the article is arranged as follows. In Section 2, we obtain the likelihood function. In Section 3, we apply the EM algorithm to calculate the MLEs. In Section 4, we compute the observed information matrix based on the missing value principle. Next, we adopt the bootstrap method to obtain the confidence intervals. The Bayesian inference is presented in Section 5. In Section 6, a Monte Carlo simulation and real data analysis are shown. In Section 7, we derive the maximum likelihood estimation and Bayesian inference of the parameters that have order restrictions.

## 2. Likelihood Function

Generate lifetime $X_{1:m:n},X_{2:m:n},\cdots$, $X_{i:m:n}$ from the GIED with the progressive type-II censoring scheme $(R_{1},\cdots$, $R_{k})$. The observed data are $x=(x_{1},x_{2},\cdots$, $x_{m})$. The likelihood function is

(4) $$\begin{array}{c} L\left(\theta ,\lambda \right)=C\prod_{i=1}^{m}f\left(x_{i}\right){\left(1-F\left(x_{i}\right)\right)}^{R_{i}} \end{array}$$

where $x_{1}<x_{2}<$⋯$<x_{m}$.

Substitute (2) and (1) for (4). Then, we can obtain the observed likelihood function:

(5) $$\begin{array}{c} L\left(\theta ,\lambda \right)=C{\left(\theta \lambda \right)}^{m}e^{-\lambda \sum_{i=1}^{m}\frac{1}{x_{i}}}\prod_{i=1}^{m}{x_{i}}^{-2}{\left(1-e^{-\frac{\lambda }{x_{i}}}\right)}^{\theta -1}\prod_{i=1}^{m}{\left[{\left(1-e^{-\frac{\lambda }{x_{i}}}\right)}^{\theta }\right]}^{R_{i}} \end{array}$$

Take the derivative of (5) to obtain the log-likelihood function.

(6) $$\begin{array}{ccc} l(\theta ,\lambda ) & \propto & m\ln\theta +m\ln\lambda -\lambda \sum_{i=1}^{m}\frac{1}{x_{i}}+\sum_{i=1}^{m}{\ln x_{i}}^{-2}+\left(\theta -1\right)\sum_{i=1}^{m}\ln(1-e^{-\frac{\lambda }{x_{i}}})\\ & & +\sum_{i=1}^{m}R_{i}\ln{\left(1-e^{-\frac{\lambda }{x_{i}}}\right)}^{\theta } \end{array}$$

Suppose $X_{1}$, ⋯, $X_{m}$ are *m* items from Line 1 that are i.i.d. GIED $\left(\theta_{1},\lambda \right)$. $Y_{1}$, ⋯, $Y_{m}$ are *n* items from Line 2 that are i.i.d. GIED $\left(\theta_{2},\lambda \right)$. For a given joint progressive type-II censoring scheme $(R_{1},\cdots$, $R_{k})$, the observed data are $(\left(w_{1},z_{1},s_{1}\right)$, ⋯, $\left(w_{k},z_{k},s_{k})\right)$. Thus, the likelihood function without the normalizing constant is

(7) $$\begin{array}{ccc} L(\theta_{1},\theta_{2},\lambda |data) & = & {\theta_{1}}^{k_{1}}{\theta_{2}}^{k_{2}}\lambda^{k}\prod_{i=1}^{k}{w_{i}}^{-2}e^{-\lambda \frac{1}{w_{i}}}{\left(1-e^{-\frac{\lambda }{w_{i}}}\right)}^{z_{i}\theta_{1}+\left(1-z_{i}\right)\theta_{2}-1}\\ & & \times \prod_{i=1}^{k}{\left(1-e^{-\frac{\lambda }{w_{i}}}\right)}^{\theta_{1}s_{i}}{\left(1-e^{-\frac{\lambda }{w_{i}}}\right)}^{\theta_{2}t_{i}} \end{array}$$

where $k_{1}=\sum_{i=1}^{k}z_{i}$, $k_{2}=\sum_{i=1}^{k}\left(1-z_{i}\right)=k-k_{1}$.

When $k_{1}=0$, $k_{2}=k$, the likelihood function becomes

$$\begin{array}{ccc} L(\theta_{1},\theta_{2},\lambda |data) & = & {\theta_{2}\lambda }^{k}\prod_{i=1}^{k}{w_{i}}^{-2}e^{-\lambda \frac{1}{w_{i}}}{\left(1-e^{-\frac{\lambda }{w_{i}}}\right)}^{\theta_{2}-1}\prod_{i=1}^{k}{\left(1-e^{-\frac{\lambda }{w_{i}}}\right)}^{\theta_{1}s_{i}}{\left(1-e^{-\frac{\lambda }{w_{i}}}\right)}^{\theta_{2}t_{i}} \end{array}$$

For $s_{i}=0$, ${\left(1-e^{-\frac{\lambda }{w_{i}}}\right)}^{\theta_{1}s_{i}}=1$. For $s_{i}\neq 0$ and a fixed λ, ${\left(1-e^{-\frac{\lambda }{w_{i}}}\right)}^{\theta_{1}s_{i}}$ is a strictly decreasing function of $\theta_{1}$ that decreases to 0. Thus, for $k_{1}=0$, fixed $\theta_{2}$ and λ, $L(\theta_{1},\theta_{2},\lambda |data)$ is a strictly decreasing function of $\theta_{2}$. Therefore, there is no maximum likelihood estimate when $k_{1}$ or $k_{2}$ equals 0. Thus, we assume that $k_{1}>0$ and $k_{2}>0$.

The log-likelihood function is:

(8) $$\begin{array}{ccc} \ln L(\theta_{1},\theta_{2},\lambda |data) & = & k_{1}\ln\theta_{1}+k_{2}\ln\theta_{2}+k\ln\lambda +\sum_{i=1}^{k}\left[\theta_{1}s_{i}\ln\left(1-e^{-\frac{\lambda }{w_{i}}}\right)+\theta_{2}t_{i}\ln\left(1-e^{-\frac{\lambda }{w_{i}}}\right)\right]\\ & & +\sum_{i=1}^{k}\left[-2\ln w_{i}-\frac{\lambda }{w_{i}}+\left(z_{i}\theta_{1}+\left(1-z_{i}\right)\theta_{2}-1\right)\ln\left(1-e^{-\frac{\lambda }{w_{i}}}\right)\right] \end{array}$$

Then, we prove the MLEs of $\theta_{1}$, $\theta_{2}$ for a given λ are unique in the following.

**Theorem** **1.** *For a fixed λ>0, if $k_{1}>0$ and $k_{2}>0$, $l_{1}\left(\theta_{1},\theta_{2}\right)=\ln L\left(\theta_{1},\theta_{2},\lambda |data\right)$ is a unimodal function of $\left(\theta_{1},\theta_{2}\right)$.*

**Proof.**  Because the Hessian matrix of $l_{1}\left(\theta_{1},\theta_{2}\right)$ is a negative definite matrix, $l_{1}\left(\theta_{1},\theta_{2}\right)$ is a concave function. Moreover, for fixed $\theta_{1}\left(\theta_{2}\right)$, when $\theta_{2}\left(\theta_{1}\right)$ tends to 0, $l_{1}\left(\theta_{1},\theta_{2}\right)$ tends to −∞. When $\theta_{2}\left(\theta_{1}\right)$ tends to ∞, $l_{1}\left(\theta_{1},\theta_{2}\right)$ tends to −∞.

For a given λ, the MLEs of $\theta_{1}$ and $\theta_{2}$ are $\hat{\theta_{1}}\left(\lambda \right)$ and $\hat{\theta_{2}}\left(\lambda \right)$. They can be written as:

(9) $$\begin{array}{c} \hat{\theta_{1}}\left(\lambda \right)=\frac{-k_{1}}{M(\lambda )}\;and\;\hat{\theta_{2}}\left(\lambda \right)=\frac{-k_{2}}{N(\lambda )} \end{array}$$

where

(10) $$\begin{array}{ccc} M(\lambda ) & = & \sum_{i=1}^{k}z_{i}\ln\left(1-e^{-\frac{\lambda }{w_{i}}}\right)+\sum_{i=1}^{k}s_{i}\ln\left(1-e^{-\frac{\lambda }{w_{i}}}\right)\\ N(\lambda ) & = & \sum_{i=1}^{k}\left(1-z_{i}\right)\ln\left(1-e^{-\frac{\lambda }{w_{i}}}\right)+\sum_{i=1}^{k}t_{i}\ln\left(1-e^{-\frac{\lambda }{w_{i}}}\right) \end{array}$$

When λ is unknown, the profile log-likelihood function of λ is

(11) $$\begin{array}{ccc} p_{1}\left(\lambda \right) & = & \ln L(\hat{\theta_{1}},\hat{\theta_{2}},\lambda |data)\\ & = & -k_{1}\ln M\left(\lambda \right)-k_{2}\ln N\left(\lambda \right)+k\ln\lambda -\sum_{i=1}^{k}\left[\frac{k_{1}}{w_{i}}+\ln\left(1-e^{-\frac{\lambda }{w_{i}}}\right)\right] \end{array}$$

Maximize (11) to obtain the MLEs of λ. Then, we prove that the MLE of λ exists and is unique in the following. □

**Theorem** **2.** *If $k_{1}>0$ and $k_{2}>0$, $p_{1}\left(\lambda \right)$ is a unimodal function of λ.*

**Proof.**  The proof is given in Appendix A. □

Thus, we can obtain that for $k_{1}>0$ and $k_{2}>0$, the MLEs of $\left(\theta_{1},\theta_{2},\lambda \right)$ are unique from Theorems 1 and 2. Next, take the partial derivatives of Equation (8) and let them equal 0. Therefore, we can calculate the MLEs of the parameters. However, the equation is nonlinear, which is cumbersome to compute the solution directly. Therefore, we propose to calculate the MLEs of $\left(\theta_{1},\theta_{2},\lambda \right)$ through the EM algorithm.

## 3. Expectation Maximization Algorithm

The expectation maximization algorithm is an iterative optimization strategy based on the MLEs of the parameters. First, we introduce the potential data. The potential data can be interpreted as the data that do not have the missing variables. If we add extra variables, it becomes simpler to process. The potential data are the lifetime of the censored samples at each failure point time.

It is assumed that at the *i*-th failure time point $w_{i}$, $U_{ij}$ is the lifetime of the *j*-th censored sample from Line 1, $V_{{ij}^{\prime }}$ is the lifetime of the $j^{\prime }$-th censored sample from Line 2 for j=1,⋯, $s_{i}$, $j^{\prime }=1,\cdots$, $t_{i}$, and i=1,⋯, *k*. The observed data are $(\left(w_{1},z_{1},s_{1}\right),\cdots$, $\left(w_{k},z_{k},s_{k})\right)$. The potential data are $U=((u_{11},\cdots$, $u_{1s_{1}}),\cdots$, $(u_{k1},\cdots$, $u_{ks_{k}}))$ and $V=((v_{11},\cdots$, $v_{1t_{1}}),\cdots$, $(v_{k1},\cdots$, $v_{kt_{k}}))$. Therefore, the complete data are $(\left(w_{1},z_{1},s_{1}\right),\cdots$, $\left(w_{k},z_{k},s_{k}\right),U,V)={data}^{*}$, which are the combination of the observed data and the potential data. The log-likelihood function based on the complete data is

(12) $$\begin{array}{ccc} \ln L(\theta_{1},\theta_{2},\lambda |{data}^{*}) & = & m\ln\theta_{1}+n\ln\theta_{2}+\left(m+n\right)\ln\lambda -2\sum_{i=1}^{k}\left(\ln w_{i}+\sum_{j=1}^{s_{i}}\ln u_{ij}+\sum_{i=1}^{k}\sum_{j^{\prime }=1}^{t_{i}}\ln v_{{ij}^{\prime }}\right)\\ & & -\lambda \sum_{i=1}^{k}\left(\frac{1}{w_{i}}+\sum_{j=1}^{s_{i}}\frac{1}{u_{ij}}+\sum_{j^{\prime }=1}^{t_{i}}\frac{1}{v_{{ij}^{\prime }}}\right)+\left(\theta_{1}-1\right)\sum_{i=1}^{k}\sum_{j=1}^{s_{i}}\ln\left(1-e^{-\frac{\lambda }{u_{ij}}}\right)\\ & & +\left(\theta_{2}-1\right)\sum_{i=1}^{k}\sum_{j^{\prime }=1}^{t_{i}}\ln\left(1-e^{-\frac{\lambda }{v_{{ij}^{\prime }}}}\right)+\sum_{i=1}^{k}\left(z_{i}\theta_{1}+\left(1-z_{i}\right)\theta_{2}-1\right)\ln\left(1-e^{-\frac{\lambda }{w_{i}}}\right) \end{array}$$

In the “E”-step, the pseudo log-likelihood function is given by

(13) $$\begin{array}{ccc} l_{s}\left(\theta_{1},\theta_{2},\lambda \right) & = & m\ln\theta_{1}+n\ln\theta_{2}+\left(m+n\right)\ln\lambda -2\sum_{i=1}^{k}\left[s_{i}E\left(\ln U_{i}|U_{i}>w_{i}\right)+t_{i}E\left(\ln V_{i}|V_{i}>w_{i}\right)\right]\\ & & -2\sum_{i=1}^{k}\ln w_{i}-\lambda \sum_{i=1}^{k}\left[\frac{1}{w_{i}}+s_{i}E\left(\frac{1}{u_{ij}}\right)+t_{i}E\left(\frac{1}{V_{i}}|V_{i}>w_{i}\right)\right]\\ & & +\left(\theta_{1}-1\right)\sum_{i=1}^{k}s_{i}E\left(\ln(1-e^{-\frac{\lambda }{U_{i}}}|U_{i}>w_{i})\right)+\left(\theta_{2}-1\right)\sum_{i=1}^{k}t_{i}E\left(\ln(1-e^{-\frac{\lambda }{V_{i}}}|V_{i}>w_{i})\right)\\ & & +\sum_{i=1}^{k}\left(z_{i}\theta_{1}+\left(1-z_{i}\right)\theta_{2}-1\right)\ln\left(1-e^{\frac{\lambda }{w_{i}}}\right) \end{array}$$

The conditional pdfs of $U_{ij}$ and $V_{{ij}^{\prime }}$ can be expressed, respectively, as

$$f_{U_{ij}|W_{i}}\left(u_{ij}|w_{i}\right)=\frac{f_{GIED}\left(u_{ij},\theta_{1},\lambda \right)}{1-F_{GIED}\left(w_{i},\theta_{1},\lambda \right)}$$

$$f_{V_{{ij}^{\prime }}|W_{i}}\left(v_{{ij}^{\prime }}|w_{i}\right)=\frac{f_{GIED}\left(v_{{ij}^{\prime }},\theta_{2},\lambda \right)}{1-F_{GIED}\left(w_{i},\theta_{2},\lambda \right)}$$

where i=1,⋯, *k*.

The expectations associated with the functions of $U_{ij}$ can be written as follows:

$$E\left(\ln U_{ij}|U_{ij}>w_{i}\right)=\int_{w_{i}}^{\infty }\frac{\theta_{1}\lambda {u_{ij}}^{-2}\ln u_{ij}e^{\frac{-\lambda }{u_{ij}}}{\left(1-e^{-\frac{\lambda }{u_{ij}}}\right)}^{\theta_{1}-1}}{{\left(1-e^{-\frac{\lambda }{u_{ij}}}\right)}^{\theta_{1}}}\;du_{ij},$$

$$E\left(\frac{1}{U_{ij}}|U_{ij}>w_{i}\right)=\int_{w_{i}}^{\infty }\frac{\theta_{1}\lambda {u_{ij}}^{-3}e^{\frac{-\lambda }{u_{ij}}}{\left(1-e^{-\frac{\lambda }{u_{ij}}}\right)}^{\theta_{1}-1}}{{\left(1-e^{-\frac{\lambda }{u_{ij}}}\right)}^{\theta_{1}}}\;du_{ij},$$

$$E\left(\ln\left(1-e^{-\frac{\lambda }{u_{ij}}}\right)|U_{ij}>w_{i}\right)=\int_{w_{i}}^{\infty }\frac{\theta_{1}\lambda {u_{ij}}^{-2}\ln(1-e^{-\frac{\lambda }{u_{ij}}}e^{\frac{-\lambda }{u_{ij}}}{\left(1-e^{-\frac{\lambda }{u_{ij}}}\right)}^{\theta_{1}-1}}{{\left(1-e^{-\frac{\lambda }{u_{ij}}}\right)}^{\theta_{1}}}\;du_{ij}.$$

Similarly, we can obtain the expectations associated with the functions of $V_{{ij}^{\prime }}$.

In the “M”-step, the estimates of $\theta_{1}$, $\theta_{2}$, and λ can be calculated by maximizing the pseudo log-likelihood function with finite iterations. $\theta_{1}$ and $\theta_{2}$ with respect to λ can be calculated by taking partial derivatives of (13), as follows:

(14) $$\hat{\theta_{1}}\left(\lambda \right)=\theta_{1}=\frac{-m}{\sum_{i=1}^{k}s_{i}E\left(\ln(1-e^{-\frac{\lambda }{U_{i}}}|U_{i}>w_{i})\right)+\sum_{i=1}^{k}z_{i}\ln\left(1-e^{-\frac{\lambda }{w_{i}}}\right)}$$

(15) $$\hat{\theta_{2}}\left(\lambda \right)=\theta_{2}=\frac{-n}{\sum_{i=1}^{k}t_{i}E\left(\ln(1-e^{-\frac{\lambda }{V_{i}}}|V_{i}>w_{i})\right)+\sum_{i=1}^{k}\left(1-z_{i}\right)\ln\left(1-e^{-\frac{\lambda }{w_{i}}}\right)}$$

Use the equations above to rewrite (13) as the function only for λ. Therefore, the three-dimensional optimization problem is transformed into a one-dimensional problem. At the *r*-th iteration, $\left({\theta_{1}}^{\left(r\right)},{\theta_{2}}^{\left(r\right)},\lambda^{\left(r\right)}\right)$ denotes the estimate of $\left(\theta_{1},\theta_{2},\lambda \right)$. For fixed $\left({\theta_{1}}^{\left(r-1\right)},{\theta_{2}}^{\left(r-1\right)}\right)$, maximize $l_{s}\left(\theta_{1},\theta_{2},\lambda \right)$ to obtain $\lambda^{\left(r\right)}$. For fixed $\left({\theta_{1}}^{\left(r-1\right)},{\theta_{2}}^{\left(r-1\right)},\lambda^{\left(r\right)}\right)$, ${\theta_{1}}^{\left(r\right)}$, ${\theta_{2}}^{\left(r\right)}$ can be obtained as follow:

(16) $${\theta_{1}}^{\left(r\right)}=\frac{-m}{\sum_{i=1}^{k}s_{i}E_{\left({\theta_{1}}^{\left(r-1\right)},{\theta_{2}}^{\left(r-1\right)},\lambda^{\left(r\right)}\right)}\left(\ln(1-e^{-\frac{\lambda }{U_{i}}}|U_{i}>w_{i})\right)+\sum_{i=1}^{k}z_{i}\ln\left(1-e^{-\frac{\lambda^{\left(q\right)}}{w_{i}}}\right)}$$

(17) $${\theta_{2}}^{\left(r\right)}=\frac{-n}{\sum_{i=1}^{k}t_{i}E_{\left({\theta_{1}}^{\left(r-1\right)},{\theta_{2}}^{\left(r-1\right)},\lambda^{\left(r\right)}\right)}\left(\ln(1-e^{-\frac{\lambda }{V_{i}}}|V_{i}>w_{i})\right)+\sum_{i=1}^{k}\left(1-z_{i}\right)\ln\left(1-e^{-\frac{\lambda^{\left(q\right)}}{w_{i}}}\right)}$$

We stop the iterations when $|{\theta_{1}}^{\left(r\right)}-{\theta_{1}}^{\left(r-1\right)}|\leq 0.0001$, $|{\theta_{2}}^{\left(r\right)}-{\theta_{2}}^{\left(r-1\right)}|\leq 0.0001$, $|\lambda^{\left(r\right)}-\lambda^{\left(r-1\right)}|\leq 0.0001$. Therefore, the MLEs of $\theta_{1}$, $\theta_{2}$, λ are ${\theta_{1}}^{\left(r\right)}$, ${\theta_{2}}^{\left(r\right)}$, $\lambda^{\left(r\right)}$.

## 4. Confidence Interval Estimation

Our interest in this section is to obtain the observed information matrix based on the missing value principle. Then, we compute the interval estimations using the bootstrap-p method.

### 4.1. Observed Fisher Information Matrix

Based on the idea of [21], we have

$$\begin{array}{c} I_{o}\left(\theta_{1},\theta_{2},\lambda \right)=mI_{1}\left(\theta_{1},\theta_{2},\lambda \right)+nI_{2}\left(\theta_{1},\theta_{2},\lambda \right)-\left(\sum_{i=1}^{k}\sum_{j=1}^{s_{i}}I_{u_{ij}|w_{i}}\left(\theta_{1},\theta_{2},\lambda \right)+\sum_{i=1}^{k}\sum_{j^{\prime }=1}^{t_{i}}I_{v_{ij^{\prime }}|w_{i}}\left(\theta_{1},\theta_{2},\lambda \right)\right) \end{array}$$

and $I_{o}$ represents the observed information matrix.

$$\begin{array}{c} I_{1}\left(\theta_{1},\theta_{2},\lambda \right)=\left[\begin{array}{ccc} {I_{11}}^{\left(1\right)} & {I_{12}}^{\left(1\right)} & {I_{13}}^{\left(1\right)}\\ {I_{21}}^{\left(1\right)} & {I_{22}}^{\left(1\right)} & {I_{23}}^{\left(1\right)}\\ {I_{31}}^{\left(1\right)} & {I_{32}}^{\left(1\right)} & {I_{33}}^{\left(1\right)} \end{array}\right],\;I_{2}\left(\theta_{1},\theta_{2},\lambda \right)=\left[\begin{array}{ccc} {I_{11}}^{\left(2\right)} & {I_{12}}^{\left(2\right)} & {I_{13}}^{\left(2\right)}\\ {I_{21}}^{\left(2\right)} & {I_{22}}^{\left(2\right)} & {I_{23}}^{\left(2\right)}\\ {I_{31}}^{\left(2\right)} & {I_{32}}^{\left(2\right)} & {I_{33}}^{\left(2\right)} \end{array}\right] \end{array}$$

where

$$\begin{array}{c} {I_{11}}^{\left(1\right)}=-E\left(\frac{\partial^{2}\ln f_{GIED}\left(x,\theta_{1},\lambda \right)}{\partial {\theta_{1}}^{2}}\right),{I_{12}}^{\left(1\right)}={I_{21}}^{\left(1\right)}={I_{22}}^{\left(1\right)}={I_{23}}^{\left(1\right)}={I_{32}}^{\left(1\right)}=0\\ {I_{13}}^{\left(1\right)}={I_{31}}^{\left(1\right)}=-E\left(\frac{\partial^{2}\ln f_{GIED}\left(x,\theta_{1},\lambda \right)}{\partial \theta_{1}\partial \lambda }\right),{I_{33}}^{\left(1\right)}=-E\left(\frac{\partial^{2}\ln f_{GIED}\left(x,\theta_{1},\lambda \right)}{\partial \lambda^{2}}\right)\\ {I_{11}}^{\left(2\right)}={I_{12}}^{\left(2\right)}={I_{13}}^{\left(2\right)}={I_{21}}^{\left(2\right)}={I_{31}}^{\left(2\right)}=0,{I_{22}}^{\left(2\right)}=-E\left(\frac{\partial^{2}\ln f_{GIED}\left(x,\theta_{2},\lambda \right)}{\partial {\theta_{2}}^{2}}\right)\\ {I_{23}}^{\left(2\right)}={I_{32}}^{\left(2\right)}=-E\left(\frac{\partial^{2}\ln f_{GIED}\left(x,\theta_{2},\lambda \right)}{\partial \theta_{2}\partial \lambda }\right),{I_{33}}^{\left(2\right)}=-E\left(\frac{\partial^{2}\ln f_{GIED}\left(x,\theta_{2},\lambda \right)}{\partial \lambda^{2}}\right). \end{array}$$

The missing observed matrices are

$$\begin{array}{c} I_{U_{ij}|W_{i}}\left(\theta_{1},\theta_{2},\lambda \right)=\left[\begin{array}{ccc} {I_{11}}^{\left(3\right)} & {I_{12}}^{\left(3\right)} & {I_{13}}^{\left(3\right)}\\ {I_{21}}^{\left(3\right)} & {I_{22}}^{\left(3\right)} & {I_{23}}^{\left(3\right)}\\ {I_{31}}^{\left(3\right)} & {I_{32}}^{\left(3\right)} & {I_{33}}^{\left(3\right)} \end{array}\right],\;I_{V_{ij^{\prime }}|W_{i}}\left(\theta_{1},\theta_{2},\lambda \right)=\left[\begin{array}{ccc} {I_{11}}^{\left(4\right)} & {I_{12}}^{\left(4\right)} & {I_{13}}^{\left(4\right)}\\ {I_{21}}^{\left(4\right)} & {I_{22}}^{\left(4\right)} & {I_{23}}^{\left(4\right)}\\ {I_{31}}^{\left(4\right)} & {I_{32}}^{\left(4\right)} & {I_{33}}^{\left(4\right)} \end{array}\right] \end{array}$$

where

$$\begin{array}{c} {I_{11}}^{\left(3\right)}=-E\left(\frac{\partial^{2}\ln f_{U_{i1}|W_{i}}\left(u_{ij}|w_{i}\right)}{\partial {\theta_{1}}^{2}}\right),{I_{12}}^{\left(3\right)}={I_{21}}^{\left(3\right)}={I_{22}}^{\left(3\right)}={I_{23}}^{\left(3\right)}={I_{32}}^{\left(3\right)}=0\\ {I_{13}}^{\left(3\right)}={I_{31}}^{\left(3\right)}=-E\left(\frac{\partial^{2}\ln f_{U_{i1}|W_{i}}\left(u_{ij}|w_{i}\right)}{\partial \theta_{1}\partial \lambda }\right),{I_{33}}^{\left(3\right)}=-E\left(\frac{\partial^{2}\ln f_{U_{i1}|W_{i}}\left(u_{ij}|w_{i}\right)}{\partial \lambda^{2}}\right) \end{array}$$

$$\begin{array}{c} {I_{11}}^{\left(4\right)}={I_{12}}^{\left(4\right)}={I_{13}}^{\left(4\right)}={I_{21}}^{\left(4\right)}={I_{31}}^{\left(4\right)}=0,{I_{22}}^{\left(4\right)}=-E\left(\frac{\partial^{2}\ln f_{V_{i1}|W_{i}}\left(v_{ij}|w_{i}\right)}{\partial {\theta_{2}}^{2}}\right)\\ {I_{23}}^{\left(4\right)}={I_{32}}^{\left(4\right)}=-E\left(\frac{\partial^{2}\ln f_{V_{i1}|W_{i}}\left(v_{ij}|w_{i}\right)}{\partial \theta_{2}\partial \lambda }\right),{I_{33}}^{\left(4\right)}=-E\left(\frac{\partial^{2}\ln f_{V_{i1}|W_{i}}\left(v_{ij}|w_{i}\right)}{\partial \lambda^{2}}\right). \end{array}$$

All the expectations expressions are given in Appendix B. For every fixed $\left(\theta_{1},\theta_{2},\lambda \right)$, the covariance matrix of the estimators is the inverse matrix of the observed information matrix.

### 4.2. Bootstrap-p Method

The asymptotic confidence interval methods are based on the law of large numbers. In many practical cases, the sample size tends to be not enough. Thus, these methods have limitations about the small sample size. Reference [22] introduced the bootstrap method to construct the confidence interval. Therefore, we suggest the percentile bootstrap method to study the parametric bootstrap confidence intervals. The steps for estimating the confidence intervals are briefly summarized as follows:

Step 1: Compute the MLEs of $\hat{\theta_{\left(1\right)}}$, $\hat{\theta_{\left(2\right)}}$ and $\hat{\lambda }$ from the joint progressively type-II censored samples.
Step 2: Utilize the same censoring scheme and generate the joint progressively type-II bootstrap censored samples $\left\{x_{11}^{*},x_{21}^{*},\cdots ,x_{n1}^{*}\right\}$.
Step 3: Calculate new MLEs of $\theta_{1}$, $\theta_{2}$, and λ, say ${\hat{\theta_{1}}}^{\left(1\right)}$, ${\hat{\theta_{2}}}^{\left(1\right)}$, and ${\hat{\lambda }}_{1}^{\left(1\right)}$, from the bootstrap samples.
Step 4: Repeat Step 2 and Step 3 until running *B* times to obtain a sequence of bootstrap estimates.
Step 5: Sort $\left({\hat{\theta_{1}}}^{\left(1\right)},{\hat{\theta_{1}}}^{\left(2\right)},\cdots ,{\hat{\theta_{1}}}^{\left(B\right)}\right)$, $\left({\hat{\theta_{2}}}^{\left(1\right)},{\hat{\theta_{2}}}^{\left(2\right)},\cdots ,{\hat{\theta_{2}}}^{\left(B\right)}\right)$, and $\left({\hat{\lambda_{1}}}^{\left(1\right)},{\hat{\lambda_{2}}}^{\left(2\right)},\cdots ,{\hat{\lambda_{B}}}^{\left(B\right)}\right)$ in ascending order, respectively.
  Then, we obtain $\left(\hat{\theta_{1(1)}},\hat{\theta_{1(2)}},\cdots ,\hat{\theta_{1(B)}}\right)$, $\left(\hat{\theta_{2(1)}},\hat{\theta_{2(2)}},\cdots ,\hat{\theta_{2(B)}}\right)$, and
  $\left(\hat{\lambda_{\left(1\right)}},\hat{\lambda_{\left(2\right)}},\cdots ,\hat{\lambda_{\left(B\right)}}\right)$.
Step 6: The 100(1−ζ)% bootstrap-p confidence intervals of $\theta_{1}$, $\theta_{2}$, and λ are
  (18) $$\begin{array}{c} \;\left({\hat{\theta }}_{1([B(\zeta /2)])},{\hat{\theta }}_{1([B(1-\zeta /2)])}\right),\left({\hat{\theta }}_{2([B(\zeta /2)])},{\hat{\theta }}_{2([B(1-\zeta /2)])}\right)and\left({\hat{\lambda }}_{\left([B(\zeta /2)]\right)},{\hat{\lambda }}_{\left([B(1-\zeta /2)]\right)}\right) \end{array}$$

## 5. Bayes Estimation

Different from traditional statistics, Bayes estimation considers the prior information about life parameters. Thus, Bayesian estimation thinks about both the data provided and the prior probability to infer the interested parameters. It makes the inference of the interested parameters more objective and reasonable.

### 5.1. Bayes Estimation

Suppose that the unknown parameters $\theta_{1}$, $\theta_{2}$, and λ have Gamma prior distributions independently.

(19) $$\begin{array}{c} \pi_{1}\left(\theta_{1}\right)=\frac{{b_{1}}^{a_{1}}}{\Gamma (a_{1})}{\theta_{1}}^{a_{1}-1}e^{-b_{1}\theta_{1}},\;\theta_{1}>0;\;a_{1},b_{1}>0 \end{array}$$

(20) $$\begin{array}{c} \pi_{2}\left(\theta_{2}\right)=\frac{{b_{2}}^{a_{2}}}{\Gamma (a_{2})}{\theta_{2}}^{a_{2}-1}e^{-b_{2}\theta_{2}},\;\theta_{2}>0;\;a_{2},b_{2}>0 \end{array}$$

(21) $$\begin{array}{c} \pi_{3}\left(\lambda \right)=\frac{d^{c}}{\Gamma (c)}\lambda^{c-1}e^{-d\lambda },\;\lambda >0;\;c,d>0 \end{array}$$

where $a_{1},a_{2},b_{1},b_{2},c,d$ are the hyper-parameters that contain the prior information.

Thus, the joint prior possibility distribution can be written as

(22) $$\begin{array}{c} \pi_{0}\left(\theta_{1},\theta_{2},\lambda \right)\propto {\theta_{1}}^{a_{1}-1}{\theta_{2}}^{a_{2}-1}\lambda^{c-1}e^{-b_{1}\theta_{1}}e^{-b_{2}\theta_{2}}e^{-d\lambda } \end{array}$$

The joint posterior probability distribution is

(23) $$\begin{array}{ccc} \pi (\theta_{1},\theta_{2},\lambda |data) & = & \frac{L(\theta_{1},\theta_{2},\lambda ,data)}{\int_{0}^{\infty }\int_{0}^{\infty }\int_{0}^{\infty }\pi_{0}\left(\theta_{1},\theta_{2},\lambda \right)L\left(\theta_{1},\theta_{2},\lambda |data\right)d\theta_{1}d\theta_{2}d\lambda }\\ & & =\frac{\pi_{0}\left(\theta_{1},\theta_{2},\lambda \right)L\left(\theta_{1},\theta_{2},\lambda |data\right)}{\int_{0}^{\infty }\int_{0}^{\infty }\int_{0}^{\infty }\pi_{0}\left(\theta_{1},\theta_{2},\lambda \right)L\left(\theta_{1},\theta_{2},\lambda |data\right)d\theta_{1}d\theta_{2}d\lambda } \end{array}$$

The denominator of $\pi (\theta_{1},\theta_{2},\lambda |data)$ is a function of the observed data. Thus, $L(\theta_{1},\theta_{2},\lambda ,data)$ and $\pi (\theta_{1},\theta_{2},\lambda |data)$ have a coefficient proportional relationship. Therefore, the joint posterior probability distribution is

(24) $$\begin{array}{ccc} \pi (\theta_{1},\theta_{2},\lambda |data) & \propto & L\left(\theta_{1},\theta_{2},\lambda ,data\right)=\pi_{0}\left(\theta_{1},\theta_{2},\lambda \right)L\left(\theta_{1},\theta_{2},\lambda |data\right)\\ & & \propto {\theta_{1}}^{a_{1}+k_{1}-1}e^{-\left(b_{1}-\sum_{i=1}^{k}\left(z_{i}+s_{i}\right)\ln\left(1-e^{-\frac{\lambda }{w_{i}}}\right)\right)\theta_{1}}\times {\theta_{2}}^{a_{2}+k_{2}-1}e^{-\left(b_{2}-\sum_{i=1}^{k}\left(1-z_{i}+t_{i}\right)\ln\left(1-e^{-\frac{\lambda }{w_{i}}}\right)\right)\theta_{2}}\\ & & \times \lambda^{k+c-1}e^{-\left(d+\sum_{i=1}^{k}\frac{1}{w_{i}}\right)\lambda }\times \frac{1}{\prod_{i=1}^{k}\left(1-e^{-\frac{\lambda }{w_{i}}}\right)} \end{array}$$

We rewrite (24) as follows:

$$\begin{array}{c} \pi \left(\theta_{1},\theta_{2},\lambda |data\right)\propto \pi \left(\theta_{1}|\lambda ,data\right)\times \pi \left(\theta_{2}|\lambda ,data\right)\times \pi \left(\lambda |data\right)\times d\left(\theta_{1},\theta_{2},\lambda \right) \end{array}$$

where

$$\begin{array}{c} \pi \left(\theta_{1}|\lambda ,data\right)∼Ga\left(a_{1}+k_{1},b_{1}-\sum_{i=1}^{k}\left(z_{i}+s_{i}\right)\ln\left(1-e^{-\frac{\lambda }{w_{i}}}\right)\right) \end{array}$$

$$\begin{array}{c} \pi \left(\theta_{2}|\lambda ,data\right)∼Ga\left(a_{2}+k_{2},b_{2}-\sum_{i=1}^{k}\left(1-z_{i}+t_{i}\right)\ln\left(1-e^{-\frac{\lambda }{w_{i}}}\right)\right) \end{array}$$

$$\begin{array}{c} \pi \left(\lambda |data\right)∼Ga\left(c+k,d+\sum_{i=1}^{k}\frac{1}{w_{i}}\right) \end{array}$$

$$\begin{array}{ccc} d(\theta_{1},\theta_{2},\lambda ) & \propto & \frac{1}{\prod_{i=1}^{k}\left(1-e^{-\frac{\lambda }{w_{i}}}\right)}\times {\left[b_{1}-\sum_{i=1}^{k}\left(z_{i}+s_{i}\right)\ln\left(1-e^{-\frac{\lambda }{w_{i}}}\right)\right]}^{-(a_{1}+k_{1})}\\ & & \times {\left[b_{2}-\sum_{i=1}^{k}\left(1-z_{i}+t_{i}\right)\ln\left(1-e^{-\frac{\lambda }{w_{i}}}\right)\right]}^{-(a_{2}+k_{2})} \end{array}$$

### 5.2. Loss Functions

In Bayesian statistics, the Bayesian estimation of a function $\phi (\theta_{1},\theta_{2},\lambda )$ is derived on a prescribed loss function. Thus, it is critical to select the loss function:

- Squared error loss function (SEL)

The SEL function is given by

(25) $$\begin{array}{c} L_{s}\left(\omega ,\hat{\omega }\right)={\left(\hat{\omega }-\omega \right)}^{2} \end{array}$$

Here, $\hat{\omega }$ is an estimate of ω.

The corresponding Bayes estimate ${\hat{\omega }}_{s}$ of ω can be obtained from

(26) $$\begin{array}{c} {\hat{\omega }}_{s}=E\left[\omega |x\right] \end{array}$$

Thus, ${\hat{\phi }\left(\theta_{1},\theta_{2},\lambda \right)}_{s}$ represents the Bayes estimation of $\phi (\theta_{1},\theta_{2},\lambda )$ under the SEL function, that is,

(27) $$\begin{array}{c} {\hat{\phi }\left(\theta_{1},\theta_{2},\lambda \right)}_{s}=\int_{0}^{\infty }\int_{0}^{\infty }\int_{0}^{\infty }\phi \left(\theta_{1},\theta_{2},\lambda \right)\pi \left(\theta_{1},\theta_{2},\lambda |data\right)d\theta_{1}d\theta_{2}d\lambda \end{array}$$

- Linex loss function (LL)

The LL function is the most universally used asymmetric loss function. The asymmetric loss function is considered more comprehensive in many respects. The linex loss function is given below:

(28) $$\begin{array}{c} L_{l}\left(\omega ,\hat{\omega }\right)=e^{h(\hat{\omega }-\omega )}-h\left(\hat{\omega }-\omega \right)-1,\;h\neq 0 \end{array}$$

where $\hat{\omega }$ is an estimate of ω and *h* stands for the sign, which presents the asymmetry.

The corresponding Bayesian estimate ${\hat{\omega }}_{l}$ of ω can be derived from

(29) $$\begin{array}{c} {\hat{\omega }}_{l}=-\frac{1}{h}\ln\left[E\left(e^{-h\omega }|x\right)\right] \end{array}$$

Then, the Bayes estimation ${\hat{\phi }\left(\theta_{1},\theta_{2},\lambda \right)}_{l}$ of $\phi (\theta_{1},\theta_{2},\lambda )$ results under the LL function in being the following form

(30) $$\begin{array}{c} {\hat{\phi }\left(\theta_{1},\theta_{2},\lambda \right)}_{l}=-\frac{1}{h}\ln\left(\int_{0}^{\infty }\int_{0}^{\infty }\int_{0}^{\infty }e^{-h\phi (\theta_{1},\theta_{2},\lambda )}\pi \left(\theta_{1},\theta_{2},\lambda |data\right)d\theta_{1}d\theta_{2}d\lambda \right) \end{array}$$

Obviously, we can see from the derivation above that the form of Bayesian estimation is the ratio of two multiple integrals. It is analytically tricky to obtain an explicit solution. Therefore, we propose the importance sampling method to obtain approximate explicit forms for the Bayesian estimation.

### 5.3. Importance Sampling Method

The importance sampling method can be applied in Bayesian estimation under different loss functions. The importance sampling method algorithm can be described briefly as follows:

Step 1: Generate λ from π(λ∣data) for the given data.
Step 2: For given λ, generate $\theta_{1}$, $\theta_{2}$ from $\pi (\theta_{1}|\lambda ,data)$ and $\pi (\theta_{2}|\lambda ,data)$, respectively.
Step 3: Repeat Step 1 and Step 2 *M* times to generate $\left(\theta_{1i},\theta_{2i},\lambda_{i}\right)$, i=1,2,⋯, *M*.
Step 4: Calculate $g(\theta_{1i},\theta_{2i},\lambda_{i})$, $d(\theta_{1i},\theta_{2i},\lambda_{i})$ and the importance weight *q*. Here,
  $$\begin{array}{c} q_{i}=\frac{d_{i}}{\sum_{j=1}^{M}d_{j}} \end{array}$$
Step 5: The estimates of $g(\theta_{1},\theta_{2},\lambda )$ under the squared error loss function and linex loss function are
  $$\begin{array}{c} {\hat{g}\left(\theta_{1},\theta_{2},\lambda \right)}_{s}=\sum_{i=1}^{M}q_{i}g\left(\theta_{1i},\theta_{2i},\lambda_{i}\right) \end{array}$$
  $$\begin{array}{c} {\hat{g}\left(\theta_{1},\theta_{2},\lambda \right)}_{l}=-\frac{1}{h}\ln\left(\sum_{i=1}^{M}q_{i}e^{-hg(\theta_{1i},\theta_{2i},\lambda_{i})}\right) \end{array}$$

Sort $g_{i}$ in ascending order to obtain $(g_{\left(1\right)},g_{\left(2\right)},\cdots$, $g_{\left(M\right)})$. The relevant $q_{i}$ is recorded as $(q_{\left(1\right)},q_{\left(2\right)},\cdots$, $q_{\left(M\right)})$. A credible interval is obtained as $\left(g_{\left(n_{1}\right)},g_{\left(n_{2}\right)}\right)$, when $n_{1},n_{2}$ satisfy

(31) $$\begin{array}{c} n_{1}<n_{2},\;n_{1},n_{2}\in \left\{1,2,\cdots ,M\right\}\;and\;\sum_{i=n_{1}}^{n_{2}}q_{i}\leq 1-\zeta <\sum_{i=n_{1}}^{n_{2}+1}q_{i} \end{array}$$

Thus, the 100(1−ζ)% symmetric credible intervals $g(\theta_{1},\theta_{2},\lambda )$ are $\left(g_{\left(\left[N\frac{\zeta }{2}\right]\right)},g_{\left(\left[N\left(1-\frac{\zeta }{2}\right)\right]\right)}\right)$.

## 6. Simulation and Data Analysis

### 6.1. Numerical Simulation

We conducted simulation experiments in an attempt to analyze the performance of different methods mentioned above. We performed this for various censoring schemes. Here, the notation $\left(0_{\left(6\right)},10_{\left(4\right)},0_{\left(10\right)}\right)$ implies $R_{1}=\cdots =R_{6}=0$,$R_{7}=\cdots =R_{10}=4$, $R_{11}=\cdots =R_{20}=0$. Let m=20,n=25, and k=20,25, and 30. Set $\theta_{1}=1,\theta_{2}=1,\lambda =0.5$ as the true values, which are considered as the initial values in the EM algorithm. The Bayesian estimates under the squared error loss function and linex loss function are calculated for informative and non-informative priors. In light of [23], the hyper-parameters for the non-informative prior is $10^{-5}$. For the informative prior, the corresponding hyper parameters are $a_{1}=2$, $b_{1}=1$, $a_{2}=1,b_{2}=2,c=3,d=2$. The linex constant are h=2. Based on the MLEs calculated by the EM algorithm, the percentile bootstrap confidence intervals can be derived. Then, we constructed the Bayesian symmetric credible intervals. IP represents informative prior and NIP represents non-informative prior.

Table 1 shows the average estimates (AEs) and the mean-squared errors (MSEs) of the MLEs for various JPC schemes based on 1000 repetitions of the EM algorithm. Table 2 and Table 3 present the Bayesian estimates and the MSEs for informative prior under the squared error loss function and the linex loss function based on 1000 repetitions, respectively. Table 4 and Table 5 show the Bayesian estimates and the MSEs for the non-informative prior under the squared error loss function and the linex loss function, which are based on 1000 repetitions, respectively. Table 6 presents the 90% percentile bootstrap confidence intervals, which contain 1000 bootstrap samples in each replication, as well as 90% symmetric credible intervals based on informative and non-informative priors. The average lengths (ALs) and the coverage percentages (CPs) of these intervals were calculated based on 1000 repetitions.

From Table 1, we can find that the MLEs perform better in terms of the MSEs as *k* increases. The MSEs of λ are always much smaller than those of $\theta_{1}$ and $\theta_{2}$, which means λ obtains better estimates. This is reasonable because λ is the same in the two populations. From Table 2 and Table 3, it is clear that the MSEs of Bayesian estimates become smaller as *k* increases. The MSEs are smaller and the AEs are closer to the true value under the linex loss function than the squared error loss function. From Table 4 and Table 5, it is found that if *k* is larger, and the results are closer to the true value. Comparing the MLEs and Bayesian estimates, Bayes inference is superior to the MLEs. Bayesian estimates with the informative prior perform better than those with the non-informative prior. Moreover, the results are better under the linex loss function. All in all, Bayesian estimates with the informative prior under the linex loss function perform best among the methods discussed. From Table 6, we can find that the ALs of the symmetric credible intervals are shorter than the bootstrap confidence intervals. The ALs of the NIP are larger than the IP. The CPs of the NIP are less than the IP. Therefore, we can conclude that the IP performs better than the NIP. Comparing these approaches, the Bayesian method obtains better results in the confidence intervals. When *k* is large, the CPs of both Bayes method and bootstrap method increase greatly. Thus, the Bayesian approach with the informative prior is superior to the other two methods.

### 6.2. Real Data Analysis

The two sets of real data were the data of the breaking strength of jute fiber. We analyzed them and applied the methods mentioned above. The data sets were from [24]. Each set has 30 observations. The data are shown in Table 7 and Table 8.

The data sets were divided by 1000 for the ease of use. We conducted the Kolmogorov–Smirnov distance test, which computes the difference between empirical distribution functions and the fit distributions. Therefore, the MLEs, the K-S distance, and the corresponding *p* values can be obtained. We found that the GIED fit well for both data sets. The results are presented in Table 9.

The likelihood ratio test was used to test if the scale parameters can be considered as the same value. $H_{0}:\lambda_{1}=\lambda_{2}$. After calculation, the *p* value was 0.688. Therefore, the null hypothesis cannot be rejected. The two scale parameters can be considered as the same. On account of the null hypothesis, the MLEs of the parameters were calculated to be $\hat{\theta_{1}}=1.454,\hat{\theta_{2}}=1.596,\hat{\lambda }=0.228$.

We generated the observed data for two censoring schemes: $\left(0_{\left(14\right)},30,0_{\left(15\right)}\right)$ and $\left(0_{\left(14\right)},15,15,0_{\left(14\right)}\right)$ from the complete data above. Thus, the MLEs of the parameters along with the associated AEs and MSEs were computed. The results are shown in Table 10.

Due to the limitation of the conditions, we cannot obtain the informative prior. Therefore, all Bayesian estimates in real data analysis were based on the non-informative prior. For the non-informative prior, the results of the Bayesian approach under the squared error loss function and the linex loss function are presented in Table 11 and Table 12, respectively. We computed the 90% confidence/credible intervals with the bootstrap-p method and Bayesian estimations. The results are shown in Table 13. Here, LB denotes lower bound and UB denotes upper bound.

## 7. Order-Restricted Inference

In many practical cases, the experimenters may know that the lifetime of diverse populations is orderly. In this section, we discuss the problem that the parameters have order restrictions. The order restriction of the scale parameter is $\theta_{1}<\theta_{2}$.

### 7.1. Maximum Likelihood Estimates

For a given λ, the function (8) is a concave function of $\theta_{1}$ and $\theta_{2}$. The maximum value is unique, and it can be obtained at the point $\left(\hat{\theta_{1}}\left(\lambda \right),\hat{\theta_{2}}\left(\lambda \right)\right)$. The order-restricted MLEs of $\theta_{1}$ and $\theta_{2}$ are $\tilde{\theta_{1}}\left(\lambda \right)$ and $\tilde{\theta_{2}}\left(\lambda \right)$, respectively. If $\hat{\theta_{1}}\left(\lambda \right)<\hat{\theta_{2}}\left(\lambda \right)$, $\tilde{\theta_{1}}\left(\lambda \right)$ equals $\hat{\theta_{1}}\left(\lambda \right)$ and $\tilde{\theta_{2}}\left(\lambda \right)$ equals $\hat{\theta_{2}}\left(\lambda \right)$. Otherwise, the maximum value of $l_{1}\left(\theta_{1},\theta_{2}\right)$ will be on the line $\theta_{1}=\theta_{2}$ under the order restriction $\theta_{1}<\theta_{2}$. Hence, we can obtain

$$\begin{array}{c} \tilde{\theta_{1}}\left(\lambda \right)=\tilde{\theta_{2}}\left(\lambda \right)=\arg\max l_{1}\left(\theta_{1},\theta_{2}\right) \end{array}$$

Therefore, for a given λ, we can obtain the following results:

(32) $$\begin{array}{c} \left(\tilde{\theta_{1}}\left(\lambda \right),\tilde{\theta_{2}}\left(\lambda \right)\right)=\left\{\begin{array}{cc} \left(\frac{k}{\sum_{i=1}^{k}\left(R_{i}+1\right)\ln\left(1-e^{-\frac{\lambda }{w_{i}}}\right)},\frac{k}{\sum_{i=1}^{k}\left(R_{i}+1\right)\ln\left(1-e^{-\frac{\lambda }{w_{i}}}\right)}\right) & \hat{\theta_{1}}\left(\lambda \right)\geq \hat{\theta_{2}}\left(\lambda \right)\\ \;\;\;(\hat{\theta_{1}}\left(\lambda \right),\hat{\theta_{2}}\left(\lambda \right)) & \hat{\theta_{1}}\left(\lambda \right)<\hat{\theta_{2}}\left(\lambda \right) \end{array}\right. \end{array}$$

Maximize $p_{2}\left(\lambda \right)=\ln L\left(\lambda ,\tilde{\theta_{1}}\left(\lambda \right),\tilde{\theta_{2}}\left(\lambda \right)|data\right)$ to obtain the MLE of λ, say $\tilde{\lambda }$. Then, we prove the MLE of λ exists and is unique.

**Theorem** **3.** *If $k_{1}>0$ and $k_{2}>0$, $p_{2}\left(\lambda \right)$ is a unimodal function of λ.*

**Proof.**  This is consistent with Theorem 2. $p_{2}\left(\lambda \right)$ is log-concave, and $p_{2}\left(\lambda \right)$ is a continuous function. □

After obtaining $\tilde{\lambda }$, we can calculate $\tilde{\theta_{1}}\left(\lambda \right)$ and $\tilde{\theta_{2}}\left(\lambda \right)$ from (32) explicitly. Therefore, we can apply the bootstrap method mentioned in Section 4 to derive the confidence intervals.

### 7.2. Bayes Estimation

Suppose $\theta_{1}<\theta_{2}$. We can have the following prior assumption based on the idea of [15]:

(33) $$\begin{array}{c} \pi_{0}^{\prime }\left(\theta_{1},\theta_{2},\lambda \right)\propto \left({\theta_{1}}^{a_{1}-1}{\theta_{2}}^{a_{2}-1}+{\theta_{1}}^{a_{2}-1}{\theta_{2}}^{a_{1}-1}\right)\lambda^{c-1}e^{-b_{1}\theta_{1}}e^{-b_{2}\theta_{2}}e^{-d\lambda } \end{array}$$

Therefore, the joint posterior probability distribution for λ>0, $0<\theta_{1}<\theta_{2}$ is

(34) $$\begin{array}{ccc} \pi^{\prime }\left(\theta_{1},\theta_{2},\lambda |data\right) & \propto & L\left(\theta_{1},\theta_{2},\lambda ,data\right)=\pi_{0}\left(\theta_{1},\theta_{2},\lambda \right)L\left(\theta_{1},\theta_{2},\lambda |data\right)\\ & & \propto \left({\theta_{1}}^{a_{1}-1}{\theta_{2}}^{a_{2}-1}+{\theta_{1}}^{a_{2}-1}{\theta_{2}}^{a_{1}-1}\right)\times {\theta_{1}}^{k_{1}}e^{-\left(b_{1}-\sum_{i=1}^{k}\left(z_{i}+s_{i}\right)\ln\left(1-e^{-\frac{\lambda }{w_{i}}}\right)\right)\theta_{1}}\\ & & \times \lambda^{k+c-1}e^{-\left(d+\sum_{i=1}^{k}\frac{1}{w_{i}}\right)\lambda }\times {\theta_{2}}^{k_{2}}e^{-\left(b_{2}-\sum_{i=1}^{k}\left(1-z_{i}+t_{i}\right)\ln\left(1-e^{-\frac{\lambda }{w_{i}}}\right)\right)\theta_{2}}\times \frac{1}{\prod_{i=1}^{k}\left(1-e^{-\frac{\lambda }{w_{i}}}\right)} \end{array}$$

Later, we can further discuss to which distribution each belongs. Therefore, the importance sampling technique mentioned in Section 5 can be conducted to derive the Bayesian inference and the credible intervals of the parameters.

More work will be performed in the future.

## 8. Conclusions

In this article, we studied the JPC scheme for two groups that follow the GIED with the same scale parameter. We adopted the EM algorithm to calculate the MLEs of the parameters. The missing value principle is important in deriving the observed information matrix. Interval estimations were obtained through the bootstrap-p method. It was assumed that the parameters have a Gamma prior. The Bayes estimates and credible intervals for the informative prior and the non-informative prior under the squared error and the linex loss function were calculated by applying the importance sampling technique. We also compared the difference of diverse methods, priors, and loss functions in the interval estimation. Moreover, in many practical cases, the experimenters may know that the lifetime of diverse populations is orderly. We investigated the problem that the parameters have order restrictions. We considered the maximum likelihood estimation and Bayesian inference of the parameters.

In the future, extensive work will be performed with more populations and different distributions. We will consider the case of different scale parameters and independent samples in the GIED.

## Figures and Tables

**Table 1:** The AEs and MSEs of the MLEs with $n=20,m=25,\theta_{1}=1,\theta_{2}=1,\lambda =0.5$.

*k*	Scheme	$\theta_{1}$		$\theta_{2}$		λ	
		AE	MSE	AE	MSE	AE	MSE
20	$\left(0_{\left(4\right)},25,0_{\left(15\right)}\right)$	1.890	0.791	0.464	0.287	0.573	0.005
	$\left(0_{\left(9\right)},25,0_{\left(10\right)}\right)$	1.812	0.659	1.582	0.338	0.584	0.007
	$\left(0_{\left(14\right)},25,0_{\left(5\right)}\right)$	0.853	0.022	0.340	0.436	0.848	0.121
	$\left(0_{\left(5\right)},5_{\left(5\right)},0_{\left(10\right)}\right)$	1.922	0.851	1.116	0.014	0.633	0.018
	$\left(25,0_{\left(19\right)}\right)$	2.091	1.190	0.716	0.081	0.597	0.009
	$\left(2_{\left(12\right)},1,0_{\left(7\right)}\right)$	1.973	0.947	0.667	0.111	0.569	0.005
25	$\left(0_{\left(4\right)},20,0_{\left(20\right)}\right)$	1.879	0.772	1.492	0.242	0.653	0.023
	$\left(0_{\left(9\right)},20,0_{\left(15\right)}\right)$	1.725	0.526	0.790	0.044	0.803	0.092
	$\left(0_{\left(14\right)},20,0_{\left(10\right)}\right)$	0.783	0.047	0.615	0.148	0.892	0.153
	$\left(0_{\left(5\right)},5_{\left(4\right)},0_{\left(16\right)}\right)$	1.486	0.236	1.693	0.480	0.412	0.008
	$\left(20,0_{\left(14\right)}\right)$	1.896	0.803	1.402	0.162	0.576	0.006
	$\left(2_{\left(10\right)},1,0_{\left(15\right)}\right)$	1.862	0.743	1.114	0.013	0.741	0.058
30	$\left(0_{\left(4\right)},15,0_{\left(25\right)}\right)$	1.825	0.681	0.764	0.056	0.643	0.020
	$\left(0_{\left(9\right)},15,0_{\left(20\right)}\right)$	1.414	0.172	0.512	0.238	0.849	0.121
	$\left(0_{\left(14\right)},15,0_{\left(15\right)}\right)$	1.219	0.048	0.683	0.101	0.622	0.015
	$\left(0_{\left(5\right)},5_{\left(6\right)},0_{\left(19\right)}\right)$	1.825	0.681	0.883	0.014	0.769	0.072
	$\left(15,0_{\left(29\right)}\right)$	1.634	0.402	1.166	0.027	0.430	0.005
	$\left(2_{\left(7\right)},1,0_{\left(22\right)}\right)$	1.676	0.457	0.815	0.034	0.647	0.022

**Table 2:** The AEs and MSEs of the Bayes estimates for the informative prior under the squared error loss function.

*k*	Scheme	$\theta_{1}$		$\theta_{2}$		λ	
		AE	MSE	AE	MSE	AE	MSE
20	$\left(0_{\left(4\right)},25,0_{\left(15\right)}\right)$	1.812	0.659	0.546	0.206	0.548	0.002
	$\left(0_{\left(9\right)},25,0_{\left(10\right)}\right)$	1.511	0.261	1.480	0.231	0.576	0.006
	$\left(0_{\left(14\right)},25,0_{\left(5\right)}\right)$	0.892	0.012	0.454	0.298	0.831	0.110
	$\left(0_{\left(5\right)},5_{\left(5\right)},0_{\left(10\right)}\right)$	1.865	0.748	1.042	0.002	0.585	0.007
	$\left(25,0_{\left(19\right)}\right)$	2.072	1.149	0.619	0.145	0.551	0.003
	$\left(2_{\left(12\right)},1,0_{\left(7\right)}\right)$	1.814	0.663	0.757	0.059	0.566	0.004
25	$\left(0_{\left(4\right)},20,0_{\left(20\right)}\right)$	1.865	0.748	1.399	0.159	0.629	0.017
	$\left(0_{\left(9\right)},20,0_{\left(15\right)}\right)$	1.645	0.416	0.849	0.023	0.742	0.059
	$\left(0_{\left(14\right)},20,0_{\left(10\right)}\right)$	0.678	0.104	0.752	0.062	0.796	0.088
	$\left(0_{\left(5\right)},5_{\left(4\right)},0_{\left(16\right)}\right)$	1.449	0.201	1.579	0.336	0.433	0.005
	$\left(20,0_{\left(14\right)}\right)$	1.883	0.780	1.320	0.103	0.547	0.002
	$\left(2_{\left(10\right)},1,0_{\left(15\right)}\right)$	1.852	0.726	1.104	0.011	0.704	0.042
30	$\left(0_{\left(4\right)},15,0_{\left(25\right)}\right)$	1.837	0.701	0.846	0.024	0.607	0.011
	$\left(0_{\left(9\right)},15,0_{\left(20\right)}\right)$	1.479	0.230	0.601	0.159	0.852	0.124
	$\left(0_{\left(14\right)},15,0_{\left(15\right)}\right)$	1.218	0.048	0.650	0.123	0.607	0.011
	$\left(0_{\left(5\right)},5_{\left(6\right)},0_{\left(19\right)}\right)$	1.873	0.762	0.928	0.005	0.747	0.061
	$\left(15,0_{\left(29\right)}\right)$	1.764	0.584	1.103	0.011	0.443	0.003
	$\left(2_{\left(7\right)},1,0_{\left(22\right)}\right)$	1.568	0.322	0.864	0.018	0.596	0.009

**Table 3:** The AEs and MSEs of the Bayes estimates for the informative prior under the linex loss function.

*k*	Scheme	$\theta_{1}$		$\theta_{2}$		λ	
		AE	MSE	AE	MSE	AE	MSE
20	$\left(0_{\left(4\right)},25,0_{\left(15\right)}\right)$	1.746	0.557	1.407	0.166	0.546	0.002
	$\left(0_{\left(9\right)},25,0_{\left(10\right)}\right)$	1.433	0.188	1.068	0.005	0.573	0.005
	$\left(0_{\left(14\right)},25,0_{\left(5\right)}\right)$	0.784	0.047	0.738	0.069	0.815	0.099
	$\left(0_{\left(5\right)},5_{\left(5\right)},0_{\left(10\right)}\right)$	1.847	0.718	1.465	0.216	0.583	0.007
	$\left(25,0_{\left(19\right)}\right)$	1.878	0.771	1.910	0.828	0.547	0.002
	$\left(2_{\left(12\right)},1,0_{\left(7\right)}\right)$	1.872	0.761	1.585	0.343	0.564	0.004
25	$\left(0_{\left(4\right)},20,0_{\left(20\right)}\right)$	1.693	0.480	2.218	1.484	0.625	0.016
	$\left(0_{\left(9\right)},20,0_{\left(15\right)}\right)$	1.541	0.292	1.994	0.988	0.737	0.056
	$\left(0_{\left(14\right)},20,0_{\left(10\right)}\right)$	0.614	0.149	0.915	0.007	0.774	0.075
	$\left(0_{\left(5\right)},5_{\left(4\right)},0_{\left(16\right)}\right)$	1.235	0.055	1.554	0.307	0.431	0.005
	$\left(20,0_{\left(14\right)}\right)$	1.894	0.799	1.976	0.952	0.541	0.002
	$\left(2_{\left(10\right)},1,0_{\left(15\right)}\right)$	2.063	1.129	1.668	0.446	0.698	0.039
30	$\left(0_{\left(4\right)},15,0_{\left(25\right)}\right)$	1.763	0.581	1.422	0.178	0.603	0.011
	$\left(0_{\left(9\right)},15,0_{\left(20\right)}\right)$	1.227	0.051	1.178	0.032	0.819	0.102
	$\left(0_{\left(14\right)},15,0_{\left(15\right)}\right)$	1.024	0.001	1.030	0.001	0.592	0.009
	$\left(0_{\left(5\right)},5_{\left(6\right)},0_{\left(19\right)}\right)$	1.984	0.969	1.299	0.090	0.744	0.059
	$\left(15,0_{\left(29\right)}\right)$	1.825	0.681	1.604	0.364	0.441	0.003
	$\left(2_{\left(7\right)},1,0_{\left(22\right)}\right)$	1.639	0.408	1.433	0.188	0.592	0.008

**Table 4:** The AEs and MSEs of the Bayes estimates for the non-informative prior under the squared error loss function.

*k*	Scheme	$\theta_{1}$		$\theta_{2}$		λ	
		AE	MSE	AE	MSE	AE	MSE
20	$\left(0_{\left(4\right)},25,0_{\left(15\right)}\right)$	2.026	1.053	0.824	0.031	0.442	0.003
	$\left(0_{\left(9\right)},25,0_{\left(10\right)}\right)$	1.338	0.114	0.363	0.406	0.462	0.001
	$\left(0_{\left(14\right)},25,0_{\left(5\right)}\right)$	0.738	0.069	0.664	0.113	0.747	0.061
	$\left(0_{\left(5\right)},5_{\left(5\right)},0_{\left(10\right)}\right)$	1.476	0.227	1.425	0.181	0.546	0.002
	$\left(25,0_{\left(19\right)}\right)$	2.038	1.078	2.003	1.006	0.558	0.003
	$\left(2_{\left(12\right)},1,0_{\left(7\right)}\right)$	2.218	1.484	0.741	0.067	0.462	0.001
25	$\left(0_{\left(4\right)},20,0_{\left(20\right)}\right)$	1.203	0.041	0.746	0.064	0.619	0.014
	$\left(0_{\left(9\right)},20,0_{\left(15\right)}\right)$	1.738	0.545	0.757	0.059	0.795	0.087
	$\left(0_{\left(14\right)},20,0_{\left(10\right)}\right)$	1.320	0.103	0.536	0.215	0.686	0.035
	$\left(0_{\left(5\right)},5_{\left(4\right)},0_{\left(16\right)}\right)$	1.526	0.276	0.919	0.006	0.570	0.005
	$\left(20,0_{\left(14\right)}\right)$	2.286	1.654	1.658	0.433	0.632	0.018
	$\left(2_{\left(10\right)},1,0_{\left(15\right)}\right)$	1.135	0.018	0.881	0.014	0.420	0.006
30	$\left(0_{\left(4\right)},15,0_{\left(25\right)}\right)$	1.622	0.387	1.130	0.017	0.633	0.018
	$\left(0_{\left(9\right)},15,0_{\left(20\right)}\right)$	1.396	0.157	1.093	0.009	0.527	0.001
	$\left(0_{\left(14\right)},15,0_{\left(15\right)}\right)$	1.443	0.196	0.738	0.069	0.569	0.005
	$\left(0_{\left(5\right)},5_{\left(6\right)},0_{\left(19\right)}\right)$	1.257	0.066	1.080	0.006	0.527	0.001
	$\left(15,0_{\left(29\right)}\right)$	1.620	0.385	0.964	0.001	0.368	0.017
	$\left(2_{\left(7\right)},1,0_{\left(22\right)}\right)$	1.879	0.773	1.580	0.336	0.454	0.002

**Table 5:** The AEs and MSEs of the Bayes estimates for the non-informative prior under the linex loss function.

*k*	Scheme	$\theta_{1}$		$\theta_{2}$		λ	
		AE	MSE	AE	MSE	AE	MSE
20	$\left(0_{\left(4\right)},25,0_{\left(15\right)}\right)$	1.546	0.298	1.553	0.305	0.441	0.004
	$\left(0_{\left(9\right)},25,0_{\left(10\right)}\right)$	1.047	0.002	0.977	0.001	0.472	0.001
	$\left(0_{\left(14\right)},25,0_{\left(5\right)}\right)$	0.661	0.115	0.841	0.025	0.725	0.051
	$\left(0_{\left(5\right)},5_{\left(5\right)},0_{\left(10\right)}\right)$	1.232	0.054	1.186	0.035	0.532	0.001
	$\left(25,0_{\left(19\right)}\right)$	1.695	0.484	1.970	0.942	0.556	0.003
	$\left(2_{\left(12\right)},1,0_{\left(7\right)}\right)$	1.643	0.414	1.423	0.179	0.473	0.001
25	$\left(0_{\left(4\right)},20,0_{\left(20\right)}\right)$	1.108	0.012	1.790	0.625	0.615	0.013
	$\left(0_{\left(9\right)},20,0_{\left(15\right)}\right)$	1.615	0.378	1.575	0.331	0.789	0.083
	$\left(0_{\left(14\right)},20,0_{\left(10\right)}\right)$	1.143	0.021	0.800	0.040	0.673	0.030
	$\left(0_{\left(5\right)},5_{\left(4\right)},0_{\left(16\right)}\right)$	1.222	0.049	1.768	0.590	0.567	0.004
	$\left(20,0_{\left(14\right)}\right)$	1.923	0.852	2.091	1.189	0.628	0.016
	$\left(2_{\left(10\right)},1,0_{\left(15\right)}\right)$	0.957	0.002	1.051	0.003	0.414	0.007
30	$\left(0_{\left(4\right)},15,0_{\left(25\right)}\right)$	1.452	0.204	1.695	0.482	0.628	0.016
	$\left(0_{\left(9\right)},15,0_{\left(20\right)}\right)$	1.233	0.054	1.248	0.061	0.522	0.001
	$\left(0_{\left(14\right)},15,0_{\left(15\right)}\right)$	1.322	0.104	0.879	0.015	0.557	0.003
	$\left(0_{\left(5\right)},5_{\left(6\right)},0_{\left(19\right)}\right)$	1.071	0.005	0.953	0.002	0.523	0.001
	$\left(15,0_{\left(29\right)}\right)$	1.420	0.177	1.635	0.403	0.365	0.018
	$\left(2_{\left(7\right)},1,0_{\left(22\right)}\right)$	1.544	0.296	1.445	0.198	0.451	0.002

**Table 6:** The interval estimations.

*k*	Scheme	Parameter	Bootstrap		IP		NIP	
			AL	CP	AL	CP	AL	CP
25	$\left(0_{\left(4\right)},20,0_{\left(20\right)}\right)$	$\theta_{1}$	2.474	85.9	1.742	88.6	2.318	86.3
		$\theta_{2}$	0.751	86.1	0.739	87.3	0.538	88.7
		λ	0.223	84.3	0.118	84.2	0.136	82.9
	$\left(0_{\left(9\right)},20,0_{\left(15\right)}\right)$	$\theta_{1}$	1.623	81.4	1.391	83.6	1.591	82.8
		$\theta_{2}$	0.884	80.6	0.932	82.1	0.663	82.6
		λ	0.167	80.3	0.142	84.7	0.163	81.2
	$\left(0_{\left(14\right)},20,0_{\left(10\right)}\right)$	$\theta_{1}$	0.981	85.2	0.914	88.1	0.946	87.4
		$\theta_{2}$	0.732	87.3	0.774	89.2	0.692	88.8
		λ	0.452	82.4	0.292	85.5	0.327	84.1
	$\left(0_{\left(5\right)},5_{\left(4\right)},0_{\left(16\right)}\right)$	$\theta_{1}$	1.638	81.5	1.508	83.8	1.688	81.9
		$\theta_{2}$	0.519	83.8	0.624	86.1	0.366	85.1
		λ	0.206	84.4	0.163	83.7	0.120	82.3
	$\left(20,0_{\left(14\right)}\right)$	$\theta_{1}$	1.547	80.4	1.508	82.8	1.598	81.5
		$\theta_{2}$	0.982	81.6	0.624	81.7	1.392	80.2
		λ	0.186	82.7	0.163	85.2	0.109	83.9
	$\left(2_{\left(10\right)},1,0_{\left(15\right)}\right)$	$\theta_{1}$	1.472	85.2	1.126	84.6	1.406	86.2
		$\theta_{2}$	1.154	87.3	0.876	85.3	1.028	88.5
		λ	0.184	82.3	0.157	85.4	0.125	83.6
30	$\left(0_{\left(4\right)},15,0_{\left(25\right)}\right)$	$\theta_{1}$	2.317	89.1	2.175	91.7	1.454	90.2
		$\theta_{2}$	1.135	90.2	0.726	92.4	1.021	91.3
		λ	0.143	88.2	0.138	87.4	0.137	86.2
	$\left(0_{\left(9\right)},15,0_{\left(20\right)}\right)$	$\theta_{1}$	1.432	84.6	1.537	86.8	1.000	85.7
		$\theta_{2}$	1.275	83.9	1.037	85.2	1.162	85.9
		λ	0.261	83.7	0.229	88.1	0.182	84.5
	$\left(0_{\left(14\right)},15,0_{\left(15\right)}\right)$	$\theta_{1}$	1.427	88.3	1.447	93.2	1.008	90.4
		$\theta_{2}$	0.982	91.5	0.675	94.3	0.913	92.1
		λ	0.518	85.4	0.256	89.6	0.516	87.3
	$\left(0_{\left(5\right)},5_{\left(6\right)},0_{\left(19\right)}\right)$	$\theta_{1}$	1.214	84.8	1.168	87.1	0.910	85.2
		$\theta_{2}$	1.036	87.1	0.938	89.2	0.590	88.4
		λ	0.213	87.5	0.218	86.8	0.145	85.6
	$\left(15,0_{\left(29\right)}\right)$	$\theta_{1}$	1.529	83.3	1.467	85.9	1.566	83.8
		$\theta_{2}$	1.325	82.8	1.316	84.7	1.026	83.6
		λ	0.248	85.7	0.228	88.3	0.115	85.2
	$\left(2_{\left(7\right)},1,0_{\left(22\right)}\right)$	$\theta_{1}$	1.993	88.4	1.632	87.1	1.986	89.5
		$\theta_{2}$	0.641	90.5	0.455	87.4	1.003	91.8
		λ	0.292	85.5	0.152	88.2	0.204	86.9

**Table 7:** Data Set 1 (Gauge length 10 mm).

Data Set 1					
43.93	50.16	101.15	108.94	123.06	141.38
151.48	163.4	177.25	183.16	212.13	257.44
262.9	291.27	303.9	323.83	353.24	376.42
383.43	422.11	506.6	530.55	590.48	637.66
671.49	693.73	700.74	704.66	727.23	778.17

**Table 8:** Data Set 2 (Gauge length 20 mm).

Data Set 2					
36.75	45.58	48.01	71.46	83.55	99.72
113.85	116.99	119.86	145.96	166.49	187.13
187.85	200.16	244.53	284.64	350.7	375.81
419.02	456.6	547.44	578.62	581.6	585.57
594.29	662.66	688.16	707.36	756.7	765.14

**Table 9:** MLEs and K-S distance.

Data Set	$\hat{\theta }$	$\hat{\lambda }$	K-S Distance	*p* Value
Data set 1	1.841	0.293	0.121	0.798
Data set 2	1.353	0.188	0.151	0.474

**Table 10:** MLEs under two schemes.

Scheme	$\hat{\theta_{1}}$	$\hat{\theta_{2}}$	$\hat{\lambda }$
$\left(0_{\left(14\right)},30,0_{\left(15\right)}\right)$	1.611	2.854	0.258
$\left(0_{\left(14\right)},15,15,0_{\left(14\right)}\right)$	1.530	2.916	0.250

**Table 11:** Bayesian estimates under the squared error loss function.

Scheme	$\hat{\theta_{1}}$	$\hat{\theta_{2}}$	$\hat{\lambda }$
$\left(0_{\left(14\right)},30,0_{\left(15\right)}\right)$	1.575	2.489	0.154
$\left(0_{\left(14\right)},15,15,0_{\left(14\right)}\right)$	1.831	2.509	0.149

**Table 12:** Bayesian estimates under the linex loss function.

Scheme	$\hat{\theta_{1}}$	$\hat{\theta_{2}}$	$\hat{\lambda }$
$\left(0_{\left(14\right)},30,0_{\left(15\right)}\right)$	1.517	2.380	0.153
$\left(0_{\left(14\right)},15,15,0_{\left(14\right)}\right)$	1.531	2.274	0.147

**Table 13:** The interval estimation of real data.

Scheme	Parameter	Bootstrap		NIP	
		LB	UB	LB	UB
$\left(0_{\left(14\right)},30,0_{\left(15\right)}\right)$	$\theta_{1}$	0.975	3.243	0.872	2.955
	$\theta_{2}$	1.113	3.947	1.102	3.768
	λ	0.104	0.341	0.096	0.329
$\left(0_{\left(14\right)},15,15,0_{\left(14\right)}\right)$	$\theta_{1}$	0.988	3.576	0.891	3.158
	$\theta_{2}$	1.016	3.825	0.963	3.549
	λ	0.091	0.324	0.086	0.314

## Data Availability

The data presented in this study are openly available in [24].

## Appendix A

Suppose $a_{j}\geq 0$, for j=1,⋯, *k*, $g\left(\lambda \right)=\sum_{i=1}^{k}a_{j}\ln\left(1-e^{-\frac{\lambda }{w_{i}}}\right)$, then −ln(g(λ)) is a concave function. The first and second derivatives of g(λ) are

$$\begin{array}{c} g^{\prime }\left(\lambda \right)=\sum_{i=1}^{k}a_{j}\frac{e^{-\frac{\lambda }{w_{i}}}}{w_{i}\left(1-e^{-\frac{\lambda }{w_{i}}}\right)}\;and\;g^{\prime \prime }\left(\lambda \right)=\sum_{i=1}^{k}a_{j}\frac{-e^{-\frac{\lambda }{w_{i}}}}{{w_{i}}^{2}{\left(1-e^{-\frac{\lambda }{w_{i}}}\right)}^{2}} \end{array}$$

Moreover,

$$\begin{array}{c} \left(\sum_{i=1}^{k}a_{j}\frac{-e^{-\frac{\lambda }{w_{i}}}}{{w_{i}}^{2}{\left(1-e^{-\frac{\lambda }{w_{i}}}\right)}^{2}}\right)\left(\sum_{i=1}^{k}a_{j}\ln\left(1-e^{-\frac{\lambda }{w_{i}}}\right)\right)-{\left(\sum_{i=1}^{k}a_{j}\frac{e^{-\frac{\lambda }{w_{i}}}}{w_{i}\left(1-e^{-\frac{\lambda }{w_{i}}}\right)}\right)}^{2}>0 \end{array}$$

Thus,

$$\begin{array}{c} -\frac{d^{2}\ln g\left(\lambda \right)}{d\lambda^{2}}=-\frac{g^{\prime \prime }\left(\lambda \right)g\left(\lambda \right)-{\left(g^{\prime }\left(\lambda \right)\right)}^{2}}{{\left(g(\lambda )\right)}^{2}}<0 \end{array}$$

Therefore, we can infer that $p_{1}\left(\lambda \right)$ is a concave function. When λ tends to 0, $p_{1}\left(\lambda \right)$ tends to −∞. When λ tends to ∞, $p_{1}\left(\lambda \right)$ tends to −∞.

## Appendix B

$$\begin{array}{cc} \; & -E\left(\frac{\partial^{2}\ln f_{GIED}\left(x,\theta_{1},\lambda \right)}{\partial {\theta_{1}}^{2}}\right)=\frac{1}{{\theta_{1}}^{2}}\\ \; & -E\left(\frac{\partial^{2}\ln f_{GIED}\left(x,\theta_{1},\lambda \right)}{\partial \theta_{1}\partial \lambda }\right)=-\int_{0}^{\infty }\theta_{1}\lambda x^{-3}e^{-\frac{2\lambda }{x}}{\left(1-e^{-\frac{\lambda }{x}}\right)}^{\theta_{1}-2}dx\\ \; & -E\left(\frac{\partial^{2}\ln f_{GIED}\left(x,\theta_{1},\lambda \right)}{\partial \lambda^{2}}\right)=-\left(\theta_{1}-1\right)\int_{0}^{\infty }\theta_{1}\lambda x^{-4}e^{-\frac{2\lambda }{x}}{\left(1-e^{-\frac{\lambda }{x}}\right)}^{\theta_{1}-3}dx+\frac{1}{\lambda^{2}}\\ \; & -E\left(\frac{\partial^{2}\ln f_{U_{i1}|W_{i}}\left(u_{ij}|w_{i}\right)}{\partial {\theta_{1}}^{2}}\right)=\frac{1}{{\theta_{2}}^{2}}\\ \; & -E\left(\frac{\partial^{2}\ln f_{U_{i1}|W_{i}}\left(u_{ij}|w_{i}\right)}{\partial \theta_{1}\partial \lambda }\right)=-\int_{w_{i}}^{\infty }\frac{\theta_{1}\lambda x^{-3}e^{-\frac{\lambda }{x}}{\left(1-e^{-\frac{\lambda }{x}}\right)}^{\theta_{1}-2}}{{\left(1-e^{-\frac{\lambda }{w_{i}}}\right)}^{\theta_{1}}}dx+\frac{e^{-\frac{\lambda }{w_{i}}}}{w_{i}\left(1-e^{-\frac{\lambda }{w_{i}}}\right)}\\ \; & -E\left(\frac{\partial^{2}\ln f_{U_{i1}|W_{i}}\left(u_{ij}|w_{i}\right)}{\partial \lambda^{2}}\right)=\theta_{1}\ln\left(1-e^{-\frac{\lambda }{w_{i}}}\right)+\frac{1}{\lambda^{2}}-\left(\theta_{1}-1\right)\int_{w_{i}}^{\infty }\frac{\theta_{1}\lambda x^{-4}e^{-\frac{\lambda }{x}}{\left(1-e^{-\frac{\lambda }{x}}\right)}^{\theta_{1}-3}}{{\left(1-e^{-\frac{\lambda }{w_{i}}}\right)}^{\theta_{1}}}dx \end{array}$$
